# Improvement of Carbonyl Groups and Surface Defects in Carbon Nanotubes to Activate Peroxydisulfate for Tetracycline Degradation

**DOI:** 10.3390/nano13010216

**Published:** 2023-01-03

**Authors:** Wenxi Wang, Junjie Zhang, Zhiran Hou, Pei Chen, Xu Zhou, Wei Wang, Fatang Tan, Xinyun Wang, Xueliang Qiao

**Affiliations:** State Key Laboratory of Materials Processing and Die & Mould Technology, School of Materials Science and Engineering, Huazhong University of Science and Technology, Wuhan 430074, China

**Keywords:** carbon nanotube, carbonyl group, surface defect, peroxydisulfate activation, singlet oxygen, tetracycline degradation

## Abstract

Carbon nanotubes (CNTs) were considered a promising activator for persulfates due to their high electrical conductivity, large specific surface area and low toxicity. The functional groups and surface defects of CNTs could significantly affect their activation performance. In this study, CNTs with high C=O ratio and defect density (CNT-O-H) were prepared through a facile treatment of raw CNTs with HNO_3_ oxidation followed by calcination at 800 °C under an argon atmosphere. X-ray photoelectron spectroscopy (XPS) and Raman results showed that the C=O proportion and defect degree (I_D_/I_G_) rose to 75% and 1.53, respectively. The obtained CNT-O-H possessed a superior performance towards peroxydisulfate (PDS) activation, and the degradation efficiency of tetracycline (TC) in the CNT-O-H/PDS system was increased to 75.2% from 56.2% of the raw CNTs/PDS system within 40 min. Moreover, the activity of CNT-O-H after use could be easily recovered with re-calcination. In addition, the CNT-O-H/PDS system exhibited high adaptabilities towards wide solution pH (2–10), common coexisting substances and diverse organic pollutants. Singlet oxygen (^1^O_2_) was confirmed to be the dominant reactive oxygen species (ROS) generated in the CNT-O-H/PDS system. It was inferred that surface C=O groups and defects of CNTs were the key site to activate PDS for TC degradation.

## 1. Introduction

Tetracycline (TC), one of the most common antibiotics, is widely used in the treatment and prevention of various diseases and infections due to its broad-spectrum antibacterial property [1,2,3]. However, TC has been frequently detected in natural aquatic environments resulting from uncontrolled discharge of pharmaceutical and hospital effluents [4,5]. Considering water solubility and recalcitrance, TC could seriously jeopardize aquatic organisms, destroy the balance of ecological system and even threaten human health via bio-concentration and food chain transmission [6,7,8]. Hence, it is of great significance to develop an effective and environmentally-friendly method to remove TC from wastewater.

Recently, advanced oxidation processes (AOPs) [9,10,11,12], particularly peroxymonosulfate (PMS) and peroxydisulfate (PDS)-based AOPs [13,14,15], have become one of the research hotspots in getting rid of refractory antibiotics due to their virtues like fast reaction rate, high degradation efficiency, no secondary contaminant and wide applicable pH range [16,17,18]. So far, a great deal of techniques, including heat [19], ultraviolet light [20], ultrasound [21], transition metal [22] and carbonaceous material [23,24], have been applied to activate PMS/PDS to produce strong oxidizing reactive oxygen species (ROS) for antibiotic degradation. Carbonaceous materials have attracted increasing attention due to their low price, nontoxicity and metal-free leaching [25,26,27,28]. Among them, carbon nanotubes (CNTs) with a high surface area, excellent electrical conductivity and special sp^2^-hybridized atom configuration have been reported to effectively activate persulfates for organic pollutant degradation [29,30].

It was reported that surface groups and defects of CNTs would remarkably affect the catalytic performance towards persulfate activation [31,32]. Several studies tried to tune the type and density of surface functional groups of CNTs in order to improve the catalytic performance [25,33]. Adil et al. [34] fabricated CNTs with surface oxygen functional groups through a repeated oxidation–reduction cycle of cobalt oxide, which displayed 6 times higher degradation rate in PMS activation to degrade 4-chlorophenol than that of primitive CNTs. After treating with concentrated H_2_SO_4_, H_3_PO_4_, KMnO_4_ and H_2_O_2_ in sequence, the CNTs could effectively activate PMS to degrade acetaminophen, and the pyridinic N and surface carbonyl groups of CNTs were dominate active sites [35]. Zhao et al. [36] used the radio-frequency Ar/O_2_ plasma technique to graft oxygen-containing functional groups (like C–O and O–C=O) onto vertically-aligned CNTs, and the O–C=O fraction could increase to around 20% from 3.7% of pristine CNTs. Additionally, the defect degree of CNTs could be adjusted by ethanol-flame treatment [37], atom doping [38] and ultrasonication in oxidative solution [39]. However, the above-mentioned modification methods suffered from tedious processes, high cost and required specific equipment, limiting their further application.

Herein, a facile strategy was developed to modify raw CNTs through a combination process of HNO_3_ oxidation and calcination at 800 °C. This method could effectively promote the C=O proportion and defect degree of CNTs, facilitating PDS activation without transition metal components and complex equipment. A series of characterization techniques including X-ray diffractometer (XRD), transmission electron microscopy (TEM), X-ray photoelectron spectroscopy (XPS), Fourier transform infrared (FT-IR) spectroscopy and Raman spectroscopy were utilized to analyze the changes of structure, morphology and chemical component of CNTs after modification. The performances of CNTs before and after treatment were comparatively evaluated by activating PDS to degrade TC. Further, the effects of activator dosage, PDS dosage, initial solution pH and temperature on TC degradation were investigated in detail. In addition, the adaptability of modified CNTs and their regeneration were explored for potential practical application. Additionally, quenching experiments and electron paramagnetic resonance (EPR) tests were conducted to determine the dominant ROS contributing to TC degradation, and an activation mechanism of modified CNTs towards PDS was proposed on the basis of XPS and EPR tests.

## 2. Materials and Methods

### 2.1. Chemical Materials

Raw CNTs (97 wt%) were purchased from Shenzhen Nanotech Port Co., Ltd. (Shenzhen, China). Melamine, Rhodamine B (RhB, 98 wt%), methyl orange (MO, 98 wt%), methyl blue (MB, 98 wt%), chlortetracycline hydrochloride (CTC, 98 wt%), sodium chloride (NaCl, 99.5 wt%), sodium bicarbonate (NaHCO_3_, 99.5 wt%), sodium nitrate (NaNO_3_, 99 wt%), ethanol (C_2_H_5_OH, 99.7 wt%), sulfuric acid (H_2_SO_4_, 95–98 wt%), nitric acid (HNO_3_, 65–68 wt%), sodium sulfate (Na_2_SO_4_, 99 wt%), hydrochloric acid (HCl, 36–38 wt%), sodium hydroxide (NaOH, 96 wt%) and *p*-benzoquinone (pBZQ, 97 wt%) were purchased form Sinopharm Chemical Reagent Co., Ltd. (Shanghai, China). Humic acid (HA, 90 wt%), tetracycline (TC, 98 wt%), potassium peroxydisulfate (PDS, 98 wt%), furfuryl alcohol (FFA,98 wt%), 4-hydroxy-TEMPO (TEMPOL, 98 wt%), 5,5-dimethyl-pyridine N-oxide (DMPO, 97 wt%) and 4-amino-2,2,6,6-tetramethylpiperidine (TEMP, 98 wt%) were provided by Aladdin Reagent Co., Ltd. (Shanghai, China). All chemicals were of analytical grade and used without any purification in the experiments.

### 2.2. Treatment of Raw CNTs 

First, 0.2 g of raw CNTs and 200 mL of nitric acid (12 M) were added to a round-bottomed glass flask and stirred at 80 °C for 48 h. After oxidation, the slurry was taken out, cooled, centrifuged and washed with water and ethanol several times until the pH was neutral. Then, the wet solid was dried at 60 °C in a vacuum oven overnight, and the collected sample was denoted as CNT-O. Further, CNT-O was heated in a tube furnace under an argon atmosphere from room temperature to 800 °C with a heating rate of 5 °C/min and kept at 800 °C for 2 h. After cooling to room temperature, the final product was obtained and labeled as CNT-O-H.

### 2.3. Characterization Methods

Phase structures were characterized by a Philips/X’Pert PRO X-ray powder diffractometer (XRD) equipped with Cu Kα radiation (λ = 0.15406 nm) (PANalytical B.V., Almelo, The Netherlands), and the morphologies of samples were explored with a Tecnai G2 20 transmission electron microscopy (TEM) with a working voltage of 200 kV (FEI company, Hillsboro, OR, USA). The composition and chemical state of samples were examined with a Kratos/Axis Ultra DLD-600 W X-ray photoelectron spectrometer (XPS, Shimadzu/Kratos company, Kyoto, Japan). The specific surface area and pore size distribution were determined with a Micromeritics ASAP2020 surface area analyzer (American Mike Instruments, Atlanta, GA, USA). The defect and disorder degree of materials were analyzed with a LabRAM HR800 Raman spectroscopy (Horiba JobinYvon, Paris, France). A Bruker VERTEX 70 Fourier transform infrared spectroscopy (FT-IR) equipped with a KBr beam splitter in the regular scanning region of 4000–400 cm^−1^ (Bruker company, Berlin, Germany) was carried out to determine chemical functional groups. The electron paramagnetic resonance (EPR) signals were detected on a Bruker X-band A200 spectrometer (Bruker company, Berlin, Germany). The electrochemical measurements were tested via a CHI660 electrochemical workstation with a standard three-electrode system (Shanghai Chenhua Instruments Co., Shanghai, China).

### 2.4. Degradation Experiment Procedure

The degradation reaction was carried out in a glass beaker containing 100 mL of 50 mg/L TC solution and a different amount of activator. The initial pH of TC solution could be adjusted using 0.1 M NaOH or H_2_SO_4_ and measured with a pH meter. Common matrix species were introduced into the initial TC solution to determine the adaptability of CNT-O-H. After magnetically stirring for 30 min to reach adsorption equilibrium, the degradation experiment was initiated by adding PDS under the corresponding conditions. At set intervals, about 3 mL of the suspension was collected and filtered through a 0.22 μm pore-size syringe in order to remove the insoluble residue. The TC concentration in solution was measured with a UV-Vis spectrometer (UV-670, Shanghai Mapada instruments Co., Ltd., Shanghai, China) at the wavelength from 280 to 700 nm. According to Lambert-Beer law, the degradation efficiency of TC was calculated by the difference between C_0_ (concentration of TC after adsorption) and C_t_ (concentration of TC at a selected time), according to the following Equation (1):

(1) $$\mathrm{Degradation}\;\mathrm{efficiency}=\frac{C_{t}-{\;C}_{0}}{C_{0}}\times 100\%$$

## 3. Results and Discussion

### 3.1. Morphological and Structural Analyses

**Figure 1:**
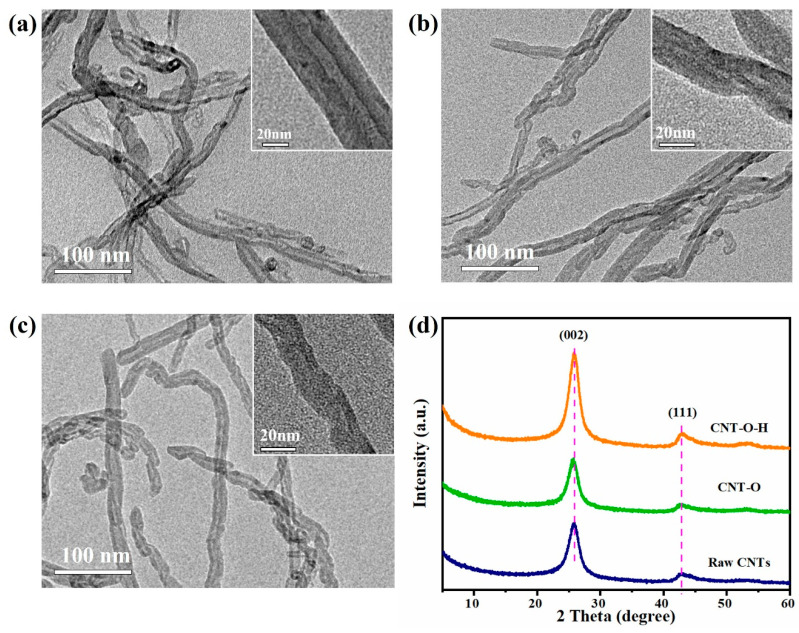
TEM images (**a**–**c**) and XRD patterns (**d**) of raw CNTs, CNT-O and CNT-O-H.

The morphologies of raw CNTs, CNT-O and CNT-O-H were examined with the TEM technique. As shown in Figure 1a–c, all samples exhibited long-tube structure with lengths from hundreds of nanometers to micrometers. Compared with the raw CNTs, CNT-O and CNT-O-H displayed shorter length and smaller diameter (Figure 1 and Figure S1), indicating the shrinking effect of acid oxidation and calcination on the length and diameter of the tubes. X-ray diffraction patterns of raw CNTs and modified CNTs samples were exhibited in Figure 1d, where the peaks at 25.9° and 42.0° could be ascribed to the (002) and (100) planes of graphite structure (JCPDS card No. 02-0212), respectively [40]. There were no extra peaks detected in the patterns, indicating the CNTs after treatment well remained the pristine crystal structure. Additionally, the peak intensities of CNT-O-H were markedly stronger than those of the other two, demonstrating a high crystallinity of CNTs obtained after the acid oxidation and calcination treatment. 

**Figure 2:**
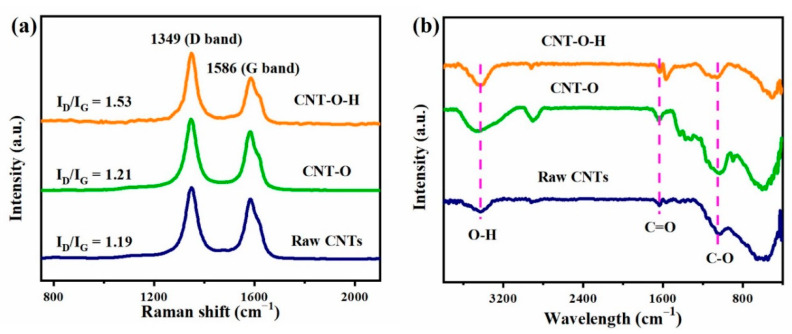
Raman spectra (**a**) and FTIR spectra (**b**) of raw CNTs, CNT-O and CNT-O-H.

The defect degrees of CNTs before and after treatment were determined with a Raman microscope. As illustrated in Figure 2a, the Raman spectra of CNTs exhibited two characteristic bands at 1349 and 1586 cm^−1^, originating from the D-(defect) and G-(graphitic) bands, respectively [41]. The ratios of the intensities of D-band to G-band (I_D_/I_G_) were estimated as 1.19, 1.21 and 1.53 for raw CNTs, CNT-O and CNT-O-H, respectively. The increase of I_D_/I_G_ ratio might be attributed to the generated defects from the decomposition of some groups at 800 °C. Clearly, the surface defects were significantly promoted in the carbon network of CNTs after heat treatment, similar to other reported results [42]. It was reported that the defects of CNTs could serve as active sites for ROS generation, which might be in favor of persulfate activation [32]. 

The functional groups of raw CNTs, CNT-O and CNT-O-H were checked with an FT-IR spectrometer and shown in Figure 2b. In comparison with raw CNTs, the intensities of C–O adsorption peak at 1048 cm^−1^ and C=O at 639 cm^−1^ remarkedly increased in CNT-O, which was attributed to the introduction of oxygen-containing groups after acid oxidation [43]. However, the intensity of the C–O peak markedly weakened while the C=O signal slightly decreased in CNT-O-H. This point suggested that more C–O groups were decomposed during the calcination process because of the relatively lower bond energy of C–O (326 kJ/mol) than that of C=O (728 kJ/mol) [44]. The broad peak at about 3430 cm^−1^ was attributed to the stretching vibration of the O–H groups, resulting from the absorbed moisture [45,46]. Apparently, a relatively strong and wide O–H signal appeared in CNT-O, owing to more hydrophilic oxygen-containing groups on the surface of CNTs. After calcination at 800 °C, the peak of O–H groups became weaker, further implying the decomposition of functional groups at high temperature.

### 3.2. BET and XPS Analyses

**Figure 3:**
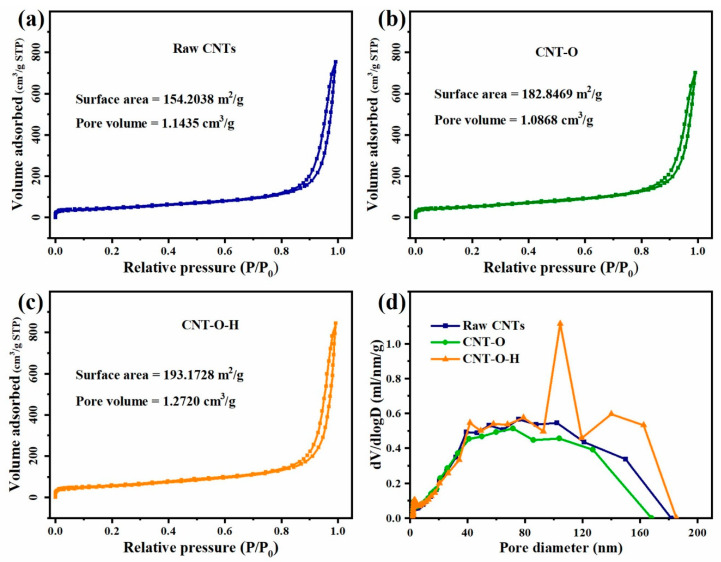
N_2_ adsorption-desorption isotherms of raw CNTs (**a**), CNT-O (**b**), CNT-O-H (**c**), and pore size distributions (**d**).

The nitrogen adsorption/desorption isotherms for raw CNTs, CNT-O and CNT-O-H were shown in Figure 3a–c, respectively, which were classified as typical type IV isotherms with a cycle of hysteresis [47]. Compared to raw CNTs and CNT-O, CNT-O-H had a larger surface area (193.2 m^2^/g) and pore volume (1.27 cm^3^/g), which would be conductive to the interaction between CNT-O-H and persulfate. The pore size distribution curves of CNTs before and after modification were calculated with the Barrett-Joyner-Halenda (BJH). As shown in Figure 3d, the average pore size for raw CNTs, CNT-O and CNT-O-H were determined about 22.59, 19.61 and 22.03 nm, respectively, indicating the mesoporous structure for all samples.

**Figure 4:**
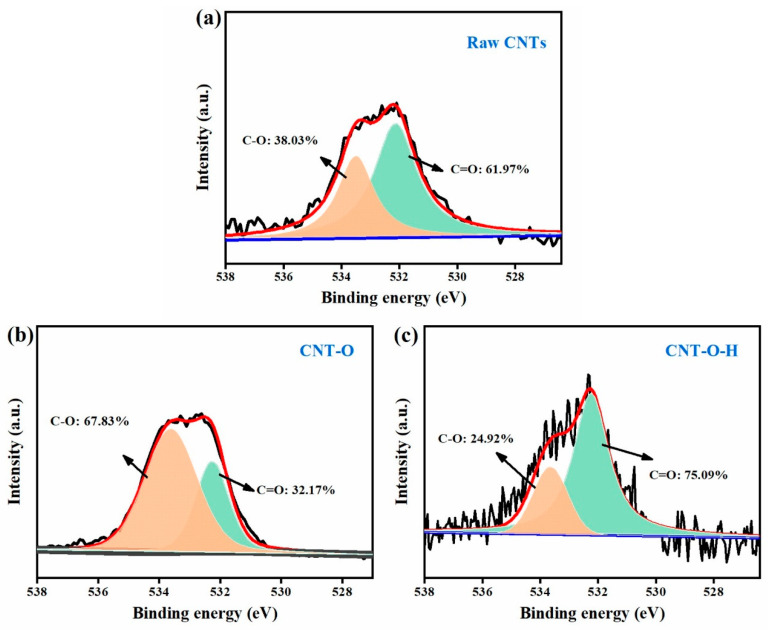
O 1s spectra of raw CNTs (**a**), CNT-O (**b**) and CNT-O-H (**c**).

Further, XPS was employed to examine the surface chemical components of raw and modified CNTs. The high-resolution C 1s spectra of all CNTs (Figure S2) could be divided into four sub-peaks positioned at 286.9, 286.0, 285.5 and 284.7 eV, which could be allocated to C=O, C–O, C–C and C=C units, respectively [48,49]. In the O 1s XPS spectra (Figure 4a–c), the peak centered at 532.2 eV corresponded to the C=O bond while the peak located at 533.6 eV was assigned to the C–O bond [50]. Interestingly, the ratios of C=O/C–O changed during the treatment process. In comparison with raw CNTs, the ratio in CNT-O decreased to 0.56 from 1.63, implying an increase of C–O groups, which was in agreement with the above FT-IR results. After calcination at 800 °C, the ratio in CNT-O-H conversely increased to 3.01, indicating a high C=O proportion in CNT-O-H. Considering that C=O could serve as active sites for persulfate activation [35], CNT-O-H with high C=O ratio was expected to possess high activity towards persulfate activation.

### 3.3. PDS Activation for Tetracycline Degradation

#### 3.3.1. Activation Abilities of Raw CNTs, CNT-O and CNT-O-H 

**Figure 5:**
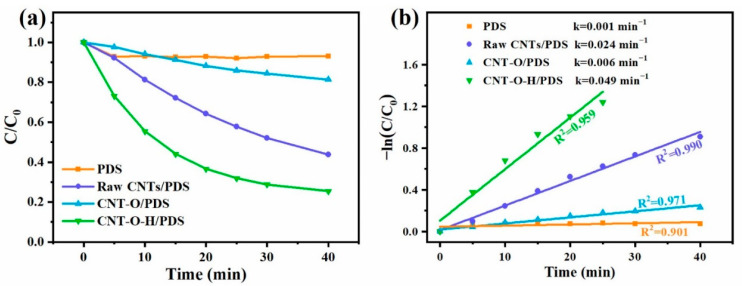
Degradation efficiencies of TC (**a**) and their pseudo-first-order kinetic fitting curves (**b**) in different systems (General conditions: 25 °C, natural pH, 0.2 g/L activator, 0.4 g/L PDS, 50 mg/L TC).

Activities of raw CNTs, CNT-O and CNT-O-H were evaluated in PDS activation for TC degradation. First, the adsorption experiments showed that the removal efficiencies of TC by raw CNTs, CNT-O and CNT-O-H were 32.2%, 33.1% and 39.8%, respectively (Figure S3a), demonstrating the better adsorption performance based on the large surface area of CNTs [51]. As shown in Figure 5a, it could be found that individual PDS removed only 6.7% of TC, showing a negligible removal ability of PDS alone. With the combination of raw CNTs, a degradation efficiency of 56.2% was observed within 40 min. However, the degradation efficiency of TC in the CNT-O/PDS system deteriorated to 18.7%, which might be attributed to the dramatically decreasing C=O ratio (Figure 4) in CNT-O compared with raw CNTs. After further heat treatment of CNT-O, the activation ability was remarkably improved, and 74.5% of TC was degraded in the CNT-O-H/PDS system. The promoting effect benefited from the significant increase of C=O ratio and defect degree.

The data of TC degradation was fitted with a pseudo-first kinetic model and pseudo-second kinetic model. It could be found that the degradation process was in better agreement with pseudo-first order model (Figure 5b) due to its higher correlation coefficients compared to the pseudo-second order model (Figure S3b). Clearly, the reaction rate constant (k) of CNT-O sharply decreased to 0.006 min^−1^, which was only a quarter of k of raw CNTs (0.024 min^−1^). After heat treatment, the k was 0.049 min^−1^ much higher than those of raw CNTs and CNT-O. In addition, the chemical oxygen demand (COD) removal rates in the CNT-O-H/PDS system were estimated to be about 44.2%, 47.9% and 54.6% at 1.5 h, 3 h and 4.5 h, respectively (Figure S4), suggesting an effective mineralization of TC in the CNT-O-H/PDS system.

#### 3.3.2. Effects of Initial pH, Temperature, CNT-O-H Dosage and PDS Dosage

**Figure 6:**
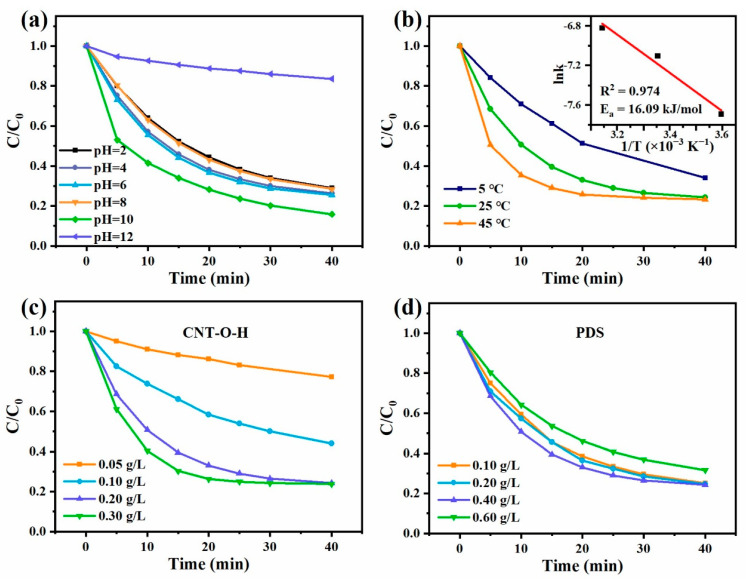
TC degradation under different initial pH (**a**), temperature (**b**), CNT-O-H dosage (**c**) and PDS dosage (**d**) (General conditions: 25 °C, natural pH, 0.2 g/L CNT-O-H, 0.4 g/L PDS, 50 mg/L TC).

The initial solution pH usually changes the surface charge of catalysts or ROS activities during the degradation reaction. Here, the influence of initial pH on TC degradation in the CNT-O-H/PDS system was studied (Figure 6a). When pH was in the range of 2–10, the degradation efficiencies of TC were higher than 70%, indicating CNT-O-H/PDS could work effectively in a wide pH scope. Notably, 84.1% of TC could be degraded within 40 min at pH 10, with the kinetic rate constant of 0.053 min^−1^ (Figure S5), demonstrating that alkaline facilitated ROS generation, whereas the degradation efficiency significantly dropped to 16.5% at pH 12. This was because excessive OH^−^ would inhibit PDS adsorption on the surface of CNTs [52].

The ability of the CNT-O-H/PDS system for TC degradation was also examined under different temperature (Figure 6b). The degradation efficiency of TC dramatically increased as the temperature raised from 5 to 45 °C. Correspondingly, the k increased from 0.030 min^−1^ at 5 °C to 0.051 min^−1^ at 45 °C. Interestingly, their k values well conformed to Arrhenius behavior (inset in Figure 6b), and the activation energy (E_a_) was calculated to be 16.09 kJ/mol, indicating that high temperature was conducive to TC degradation in the CNT-O-H/PDS system.

From Figure 6c, it could be discovered that the degradation efficiency of TC was gradually promoted with increasing the amount of CNT-O-H. When the CNT-O-H dosage was more than 0.2 g/L, the degradation efficiency slightly increased. As shown in Figure 6d, the degradation efficiency of TC could reach over 75% within 40 min when PDS concentration was ranging from 0.1 to 0.4 g/L. When the PDS dosage increased to 0.6 g/L, the degradation efficiency was conversely reduced to a certain extent. Thus, taking into account of cost and efficiency, 0.2 g/L CNT-O-H and 0.4 g/L PDS were suitable for TC degradation.

#### 3.3.3. Adaptability and Reusability

**Figure 7:**
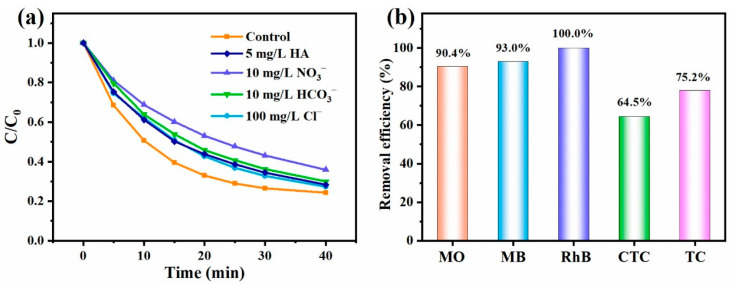
TC degradation with various matrix species (**a**) and degradation of different organic pollutants (**b**) by the CNT-O-H/PDS system (General conditions: 25 °C, natural pH, 0.2 g/L CNT-O-H, 0.4 g/L PDS, 50 mg/L TC/CTC, 20 mg/L MO/RhB, 10 mg/L MB).

The adaptability of CNT-O-H/PDS system was explored using common matrix species and different organic pollutants. First, 100 mg/L Cl^−^, 10 mg/L ${\mathrm{HCO}}_{3}^{-}$, 10 mg/L ${\mathrm{NO}}_{3}^{-}$ and 5 mg/L HA were selected as matrix species to study their influences on TC degradation in the CNT-O-H/PDS system. Obviously, the introduction of these matrix species inhibited TC degradation to a certain extent (Figure 7a), accompanying with the decrease of k value (Figure S6). A relatively strong inhibitory effect of HA was found, resulting from the competitive degradation between HA and TC. In the presence of common inorganic anions, the degradation efficiencies of TC still reached over 75%, indicating strong resistance to common matrix species. 

In addition to TC, the degradation performances of the CNT-O-H/PDS system towards MO, MB, RhB and CTC were investigated. As illustrated in Figure 7b, the removal efficiencies of MO, MB, RhB and CTC achieved 90.4%, 93.0%, 100% and 64.5%, respectively. Clearly, the CNT-O-H/PDS system exhibited high adaptabilities to various matrix species and different organic pollutants.

**Figure 8:**
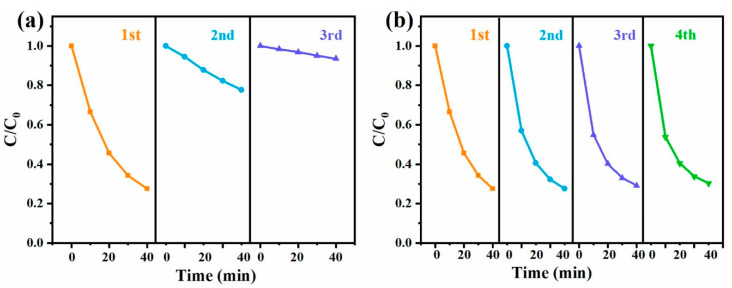
Reusability of CNT-O-H without regeneration (**a**) and with regeneration (**b**) of heat treatment (General conditions: 25 °C, natural pH, 0.2 g/L CNT-O-H, 0.4 g/L PDS, 50 mg/L TC).

In practical application, the reusability and regeneration of activators were also key factors for their use. After the degradation reaction, the precipitates were collected with centrifugation, washing and drying at 60 °C. The reusability of CNT-O-H was estimated by adding collected CNT-O-H into fresh TC solution (100 mL, 50 mg/L) after every run. As depicted in Figure 8a, the degradation efficiency sharply decreased with the reuse of CNT-O-H without regeneration. To explore the reason, the used CNT-O-H were characterized with the XRD, Raman and XPS techniques. As shown in Figure S7a, the XRD pattern of used CNT-O-H rarely changed in comparison with fresh CNT-O-H, implying no change in the crystal structure. Nevertheless, the I_D_/I_G_ value dropped significantly from 1.53 of fresh CNT-O-H (Figure 2a) to 1.11 of used CNT-O-H (Figure S7b), indicating a decrease of surface defect after use. Moreover, the C=O ratio decreased from 75% of fresh sample (Figure 4c) to 48% of used sample (Figure S7c). The results firmly proved the crucial roles of surface defect and C=O ratio of CNT-O-H in PDS activation. 

To regain the activation ability of CNT-O-H, the collected CNT-O-H was re-calcined at 800 °C under Ar atmosphere. Surprisingly, the TC degradation efficiency of regenerated CNT-O-H reverted to over 70%, even recycling for four runs. Based on above results, CNT-O-H possessed high regeneration ability and reusability after a facile re-calcination process to recover its activation performance.

### 3.4. Identification and Generation of ROS

**Figure 9:**
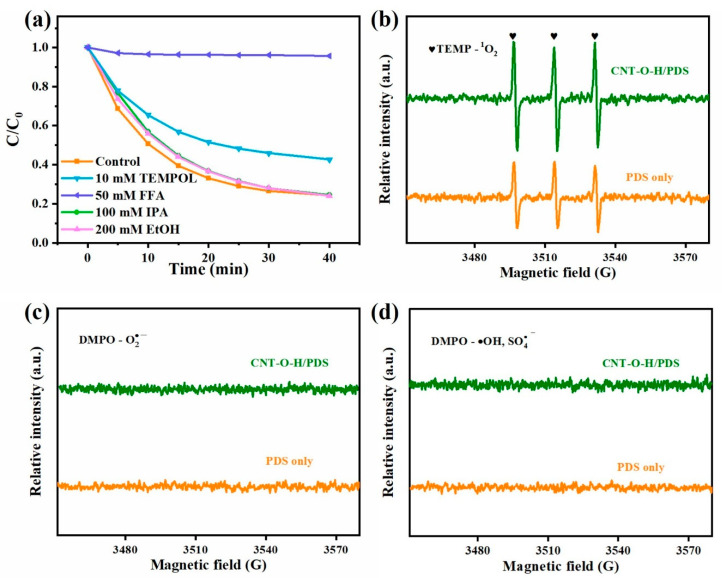
Quenching experiments (**a**) and EPR spectra (**b**–**d**) for the CNT-O-H/PDS system.

Aiming to identify the probable ROS generated in the CNT-O-H/PDS system, quenching experiments were conducted under a set of different chemical quenchers (Figure 9a). It could be observed that IPA and EtOH (probe chemicals to quench ${\mathrm{SO}}_{4}^{•-}$ and •OH) could rarely affect TC degradation, demonstrating the minimal contribution of ${\mathrm{SO}}_{4}^{•-}$ and •OH to TC degradation. The addition of TEMPOL as scavenger of $O_{2}^{•-}$ had a weak inhibitory effect on TC degradation, suggesting that $O_{2}^{•-}$ might play a minor role in TC degradation. Noticeably, the introduction of FFA could significantly inhibit TC degradation in the CNT-O-H/PDS system, demonstrating ^1^O_2_ was the main ROS responding for TC degradation. 

Further, EPR analyses were performed to confirm $O_{2}^{•-}$ and ^1^O_2_ generated in the CNT-O-H/PDS system. As presented in Figure 9b, the triplet signals related to TEMP-^1^O_2_ were observed in the CNT-O-H/PDS system, and the signal intensities were much higher than those of individual PDS. This verified that ^1^O_2_ generated in the CNT-O-H/PDS system, which was consistent with the results of quenching experiments. While no obvious $O_{2}^{•-}$ and •OH/${\mathrm{SO}}_{4}^{•-}$ signals were found in Figure 9c,d, suggesting the weak/no contribution to TC degradation. Combined with the quenching experiments, it could be inferred that the TC degradation reaction was mainly a non-radical process while ^1^O_2_ was the dominant ROS accompanied with a small amount of $O_{2}^{•-}$.

**Figure 10:**
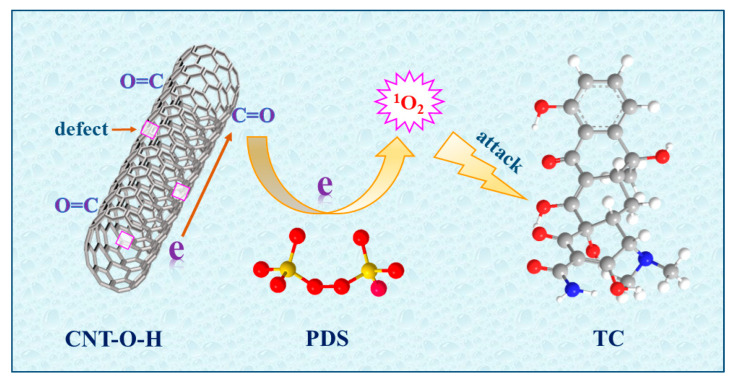
Schematic diagram for TC degradation in the CNT-O-H/PDS system.

Although persulfates could self-decomposition to form ROS [53], this function of individual PDS was negligible for TC degradation (Figure 5a). The dominant ROS generated for TC degradation was attributed to the interaction between PDS and the active sites of CNTs. As mentioned above, the combination of acid and calcination could result in high defect degree (Figure 2a) and C=O ratio (Figure 4). The surface defects of CNTs would enhance the electron transfer and adsorption of PDS molecules on CNTs [32,54]. On the other hand, the high C=O ratio of CNTs would easily transfer electrons to PDS molecules, facilitating the activation of PDS via the scission of O–O bond [32,55]. In addition, the impedance of CNTs after the combination treatment significantly reduced (Figure S8), demonstrating a favorable transmission of electrons in CNT-O-H. On the basis of the above discussion and analyses, the possible mechanism of TC degradation in the CNT-O-H/PDS system was schematically illustrated in Figure 10.

## 4. Conclusions

To sum up, the CNTs with high C=O ratio and defect degree (CNT-O-H) were successfully obtained via treating raw CNTs with nitric acid oxidation and calcination at 800 °C, which exhibited an enhanced performance towards PDS activation; 0.2 g/L CNT-O-H and 0.4 g/L PDS could degrade 75.2% of 50 mg/L TC at room temperature within 40 min. More importantly, The CNT-O-H/PDS system had strong anti-interference ability to common matrix species (100 mg/L Cl^−^, 10 mg/L ${\mathrm{HCO}}_{3}^{-}$, 10 mg/L ${\mathrm{NO}}_{3}^{-}$ and 5 mg/L HA) and high adaptability to initial solution pH range (2–10) and various organic pollutants. Moreover, the activity of used CNT-O-H was easily recovered for reuse via re-calcination. The main ROS for TC degradation was revealed to be ^1^O_2_, resulting from the interaction between PDS and active sites (C=O groups and surface defects) of CNT-O-H. This study provided a simple strategy to prepare CNTs with high C=O ratio and defect degree for persulfate activation, which possessed great potential in the practical treatment of organic-polluted wastewater.

## Data Availability

Not applicable.

## Supplementary Materials

The following supporting information can be downloaded at: https://www.mdpi.com/article/10.3390/nano13010216/s1. Figure S1: TEM images and corresponding diameter distributions of raw CNTs (a,b), CNT-O (c,d) and CNT-O-H (e,f); Figure S2: C 1 s spectra of raw CNTs (a), CNT-O (b) and CNT-O-H (c); Figure S3: TC adsorption over CNTs, CNT-O and CNT-O-H (a) and pseudo-second-order kinetic fitting curves of TC degradation in different systems (b); Figure S4: COD removal rates of CNT-O-H/PDS system at different time; Figure S5: First-order kinetic curves of TC degradation under different initial pH (a), temperature (b), CNT-O-H dosage (c) and PDS dosage (d); Figure S6: First-order kinetic curves of TC degradation with matrix species; Figure S7: XRD pattern (a), Raman spectrum (b) and O 1s spectrum (c) of used CNT-O-H; Figure S8: Impedances of raw CNTs, CNT-O and CNT-O-H.
