# Supplementary material for: Improvement of Carbonyl Groups and Surface Defects in Carbon Nanotubes to Activate Peroxydisulfate for Tetracycline Degradation
Source: Nanomaterials (Basel). 2023 Jan 3;13(1):216. doi: 10.3390/nano13010216 (PMC9824654; doi:10.3390/nano13010216)
Supplement: Supplementary file 1 [file nanomaterials-13-00216-s001.zip › nanomaterials-2109100-supplementary.pdf]

nanomaterials

Supplementary Materials

Improvement of Carbonyl Groups and Surface Defects in Carbon Nanotubes to Activate Peroxydisulfate for Tetracycline Degradation

Wenxi Wang, Junjie Zhang, Zhiran Hou, Pei Chen, Xu Zhou, Wei Wang *, Fatang Tan, Xinyun Wang and Xueliang Qiao

State Key Laboratory of Materials Processing and Die & Mould Technology, School of Materials Science and Engineering, Huazhong University of Science and Technology, Wuhan 430074, China

* Correspondence: weiwang@hust.edu.cn; Tel./Fax: +86-27-87541540

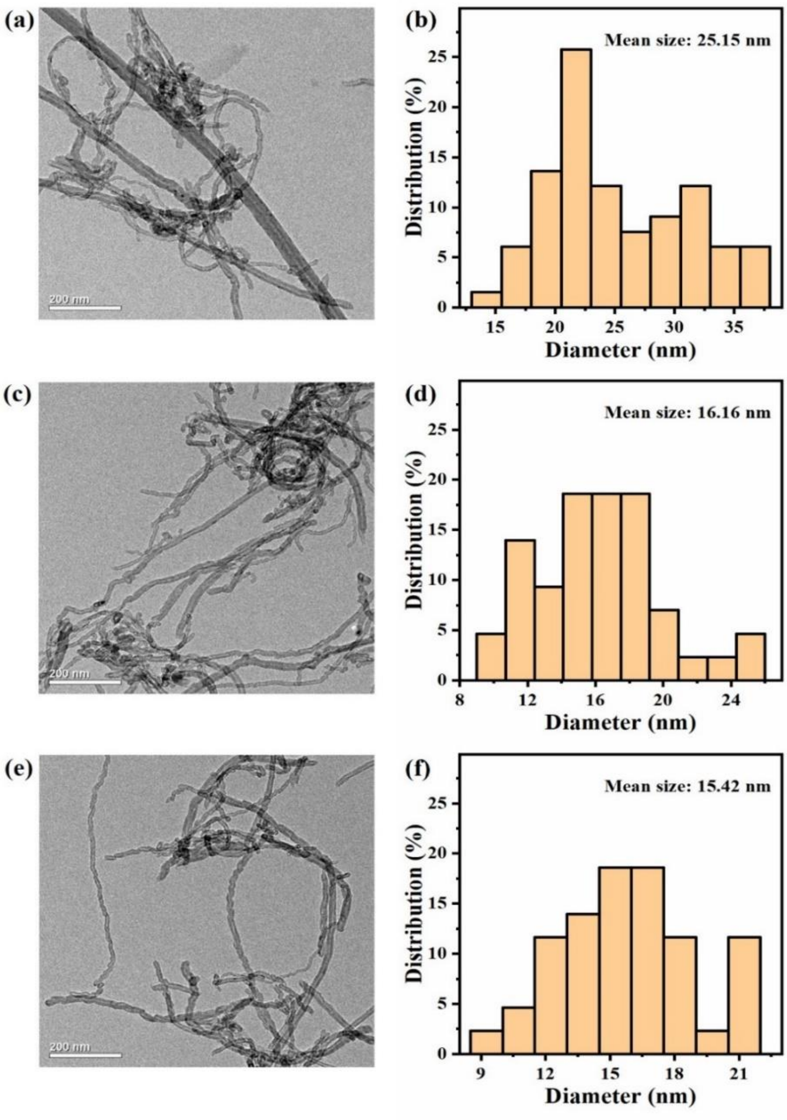

Figure S1. TEM images and corresponding diameter distributions of raw CNTs (**a**,**b**), CNT-O (**c**,**d**) and CNT-O-H (**e**,**f**).

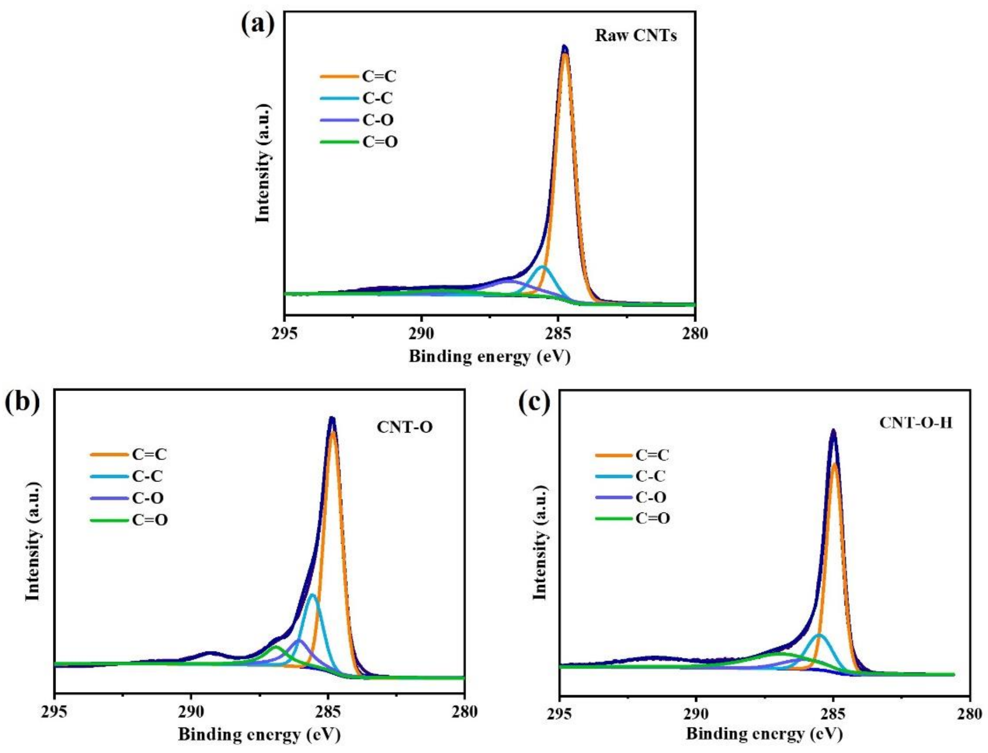

Figure S2. C 1 s spectra of raw CNTs (**a**), CNT-O (**b**) and CNT-O-H (**c**).

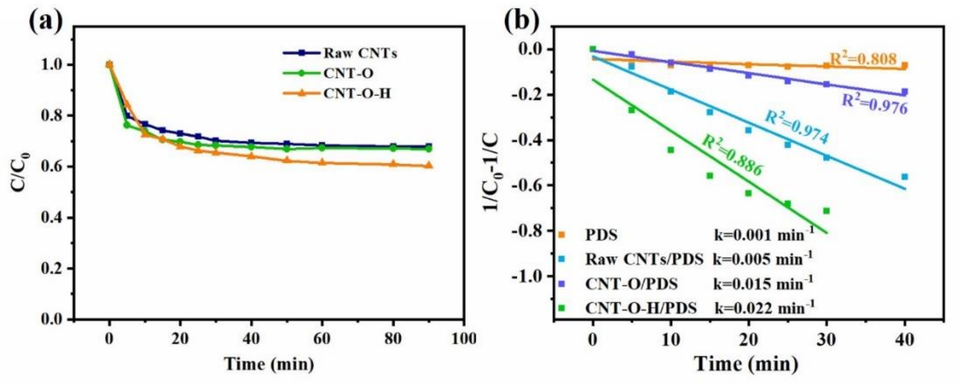

Figure S3. TC adsorption over CNTs, CNT-O and CNT-O-H (**a**) and pseudo-second-order kinetic fitting curves of TC degradation in different systems (**b**).

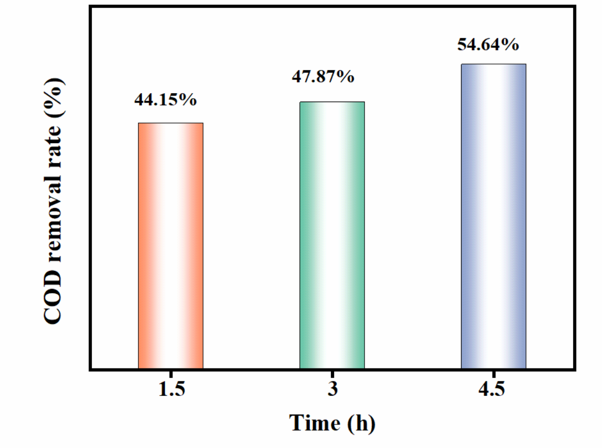

Figure S4. COD removal rates of CNT-O-H/PDS system at different time.

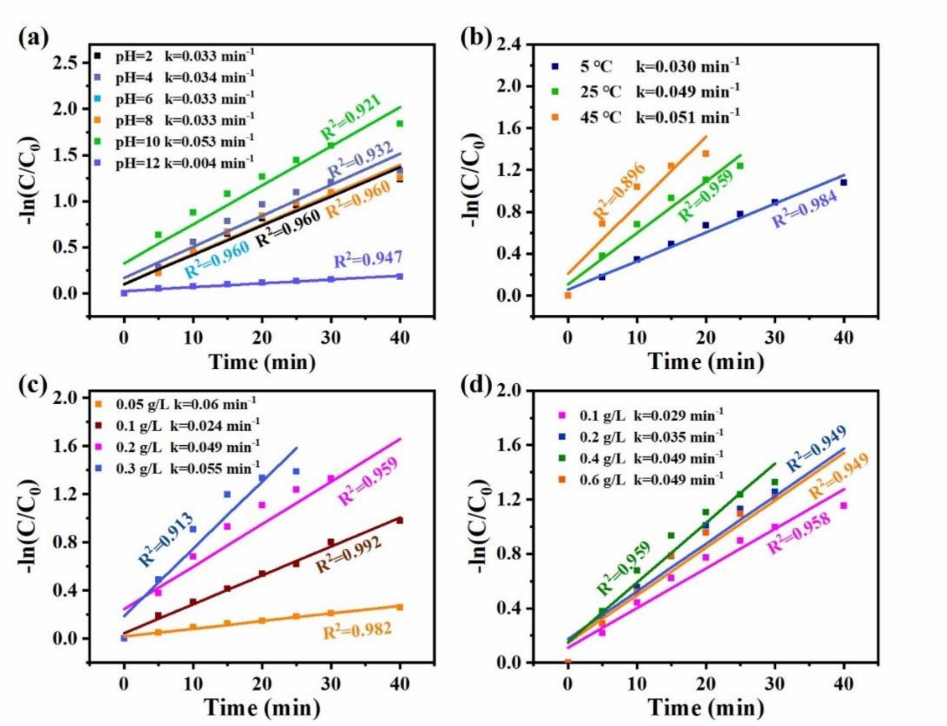

Figure S5. First-order kinetic curves of TC degradation under different initial pH (**a**), temperature (**b**), CNT-O-H dosage (**c**) and PDS dosage (**d**).

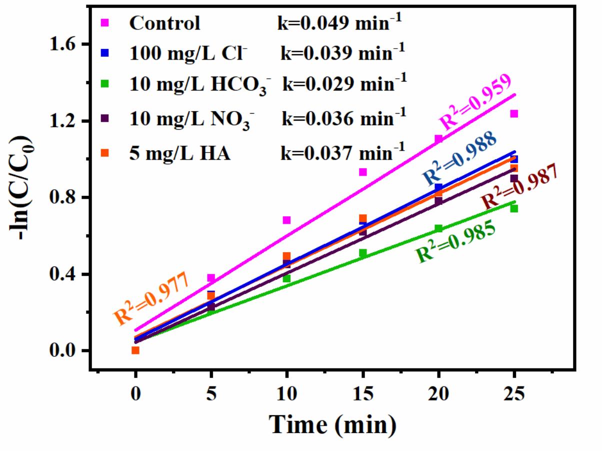

Figure S6. First-order kinetic curves of TC degradation with matrix species.

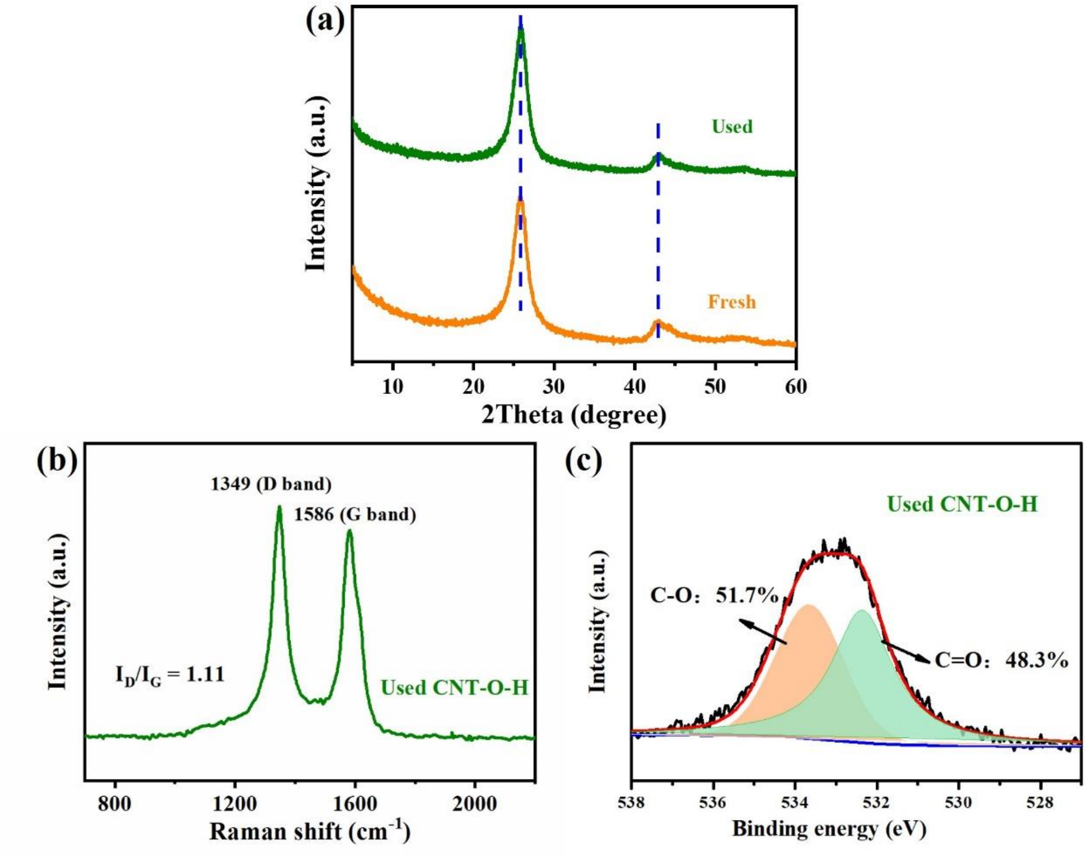

Figure S7. XRD pattern (**a**), Raman spectrum (**b**) and O 1s spectrum (c) of used CNT-O-H.

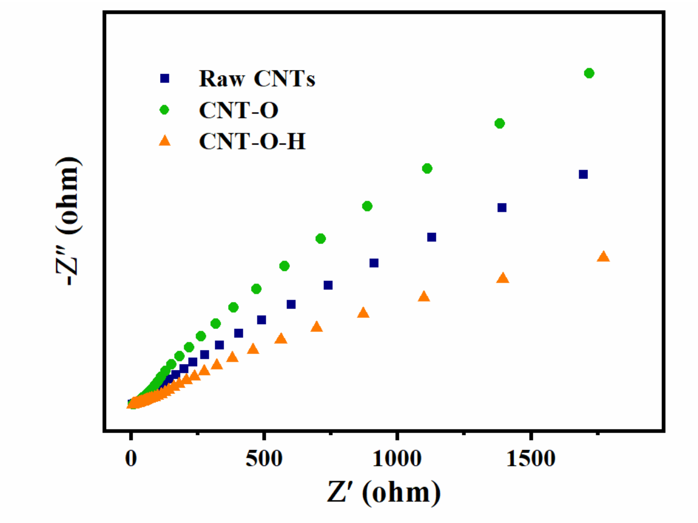

Figure S8. Impedances of raw CNTs, CNT-O and CNT-O-H.
